# Primary Cardiac Lymphoma Mimicking Cardiac Tumor: A Rare Case of Right Ventricular Mass With Seven-Year Remission

**DOI:** 10.7759/cureus.76864

**Published:** 2025-01-03

**Authors:** Vasileios Leivaditis, Athanasios Papatriantafyllou, Ioannis Panagiotopoulos, Manfred Dahm, Vasileios Lozos

**Affiliations:** 1 Department of Cardiothoracic and Vascular Surgery, Westpfalz-Klinikum, Kaiserslautern, DEU; 2 Department of Cardiac Surgery, Ippokrateio General Hospital, Athens, GRC

**Keywords:** cardiopulmonary bypass, diffuse large b-cell lymphoma, immune thrombocytopenia, primary cardiac lymphoma, right ventricular mass, tumor debulking

## Abstract

Primary cardiac lymphoma (PCL) is an exceedingly rare extranodal lymphoma type that typically presents with nonspecific symptoms such as arrhythmias or heart failure. This report details the case of a 67-year-old male with a right ventricular mass, ultimately diagnosed as diffuse large B-cell lymphoma (DLBCL) of the germinal center B-cell (GCB) subtype. Initial chemotherapy with the CHOP (cyclophosphamide, doxorubicin, vincristine, and prednisone) regimen was insufficient, necessitating the addition of rituximab and subsequent mycophenolate therapy to manage immune thrombocytopenia. Through prolonged treatment and management of complications, the patient achieved remission and has remained disease-free over a seven-year follow-up period. This case highlights the diagnostic and therapeutic challenges of PCL, especially when associated with immune thrombocytopenia and suspected sternal osteomyelitis, underlining the importance of a multidisciplinary approach.

## Introduction

Primary cardiac lymphoma (PCL) is a rare subtype of non-Hodgkin lymphoma, constituting less than 2% of primary cardiac tumors and predominantly affecting right heart structures [1]. The majority of PCL cases are diffuse large B-cell lymphomas (DLBCLs), which often present with nonspecific symptoms such as arrhythmias, heart failure, or superior vena cava syndrome, complicating timely diagnosis [2]. Imaging techniques like 2D echocardiography, cardiac magnetic resonance imaging (CMRI), and positron emission tomography-computed tomography (PET-CT) play a pivotal role in diagnosis, staging, and treatment planning for these cases [3]. Here, we present a case of primary cardiac DLBCL in a 67-year-old male, initially manifesting as a right ventricular mass with immune thrombocytopenia. The patient’s course was further complicated by febrile episodes potentially related to sternal osteomyelitis, illustrating the complexities involved in managing PCL.

## Case presentation

A 67-year-old male presented to the emergency department in July 2018 after a presyncopal episode accompanied by recurrent premature ventricular contractions. The patient also reported experiencing heart palpitations over the past two months. On admission, immune thrombocytopenia (ITP) was noted, with platelet counts between 10,000 and 20,000/μL (Table 1), prompting his admission to the hospital's cardiology department for further evaluation. His past medical history included aortic aneurysm, hypothyroidism, dyslipidemia, and hypertension.

**Table 1:** Baseline and discharge laboratory values of the patient with normal reference ranges.

Parameter	On arrival	At discharge	Normal range
Hemoglobin (g/dL)	11.2	13.7	13.5–17.8
Hematocrit (%)	33.4	41.1	40.0–53.0
Red blood cells (RBC) (×10⁶/µL)	4.1	4.6	4.4–5.9
White blood cells (×10³/µL)	8.6	6.8	3.8–10.3
Mean corpuscular volume (MCV) (fL)	88.3	90.6	80.0–96.0
Mean corpuscular hemoglobin (MCH) (pg)	29.2	31.4	28.0–33.0
Mean corpuscular hemoglobin concentration (MCHC) (g/dL)	33.1	34.3	33.0–36.0
Platelets (×10³/µL)	18.3	139.7	146–328
Sodium (mmol/L)	138	141	135–145
Potassium (mmol/L)	3.9	4.2	3.5–5.0
Calcium (mg/dL)	2.13	2.19	2.15–2.5
Urea (mg/dL)	32.8	25.2	16.6–48.5
Creatinine (mg/dL)	1.1	0.9	0.7–1.2
Glomerular filtration rate (GFR) (mL/min/1.73 m²)	75.3	90.8	>90
Aspartate aminotransferase (AST) (U/L)	48	33	10–50
Alanine aminotransferase (ALT) (U/L)	67	41	10–50
Gamma-glutamyl transferase (GGT) (U/L)	64	51	<60
Alkaline phosphatase (ALP) (U/L)	110.2	90.4	40–129
Bilirubin, total (mg/dL)	1.4	0.8	0.1–1.2
Lactate dehydrogenase (LDH) (U/L)	318	222	>250
Creatine kinase (CK) (U/L)	158	91	20–200
Total protein (g/dL)	6.1	7.6	6.6–8.7
Glucose (mg/dL)	103	95	60–99
Albumin (g/dL)	3.2	4.0	3.5–5.0
Fibrinogen (mg/dL)	447	302	193–412
C-reactive protein (CRP) (mg/L)	26.3	13.1	<5.0

**Figure 1:**
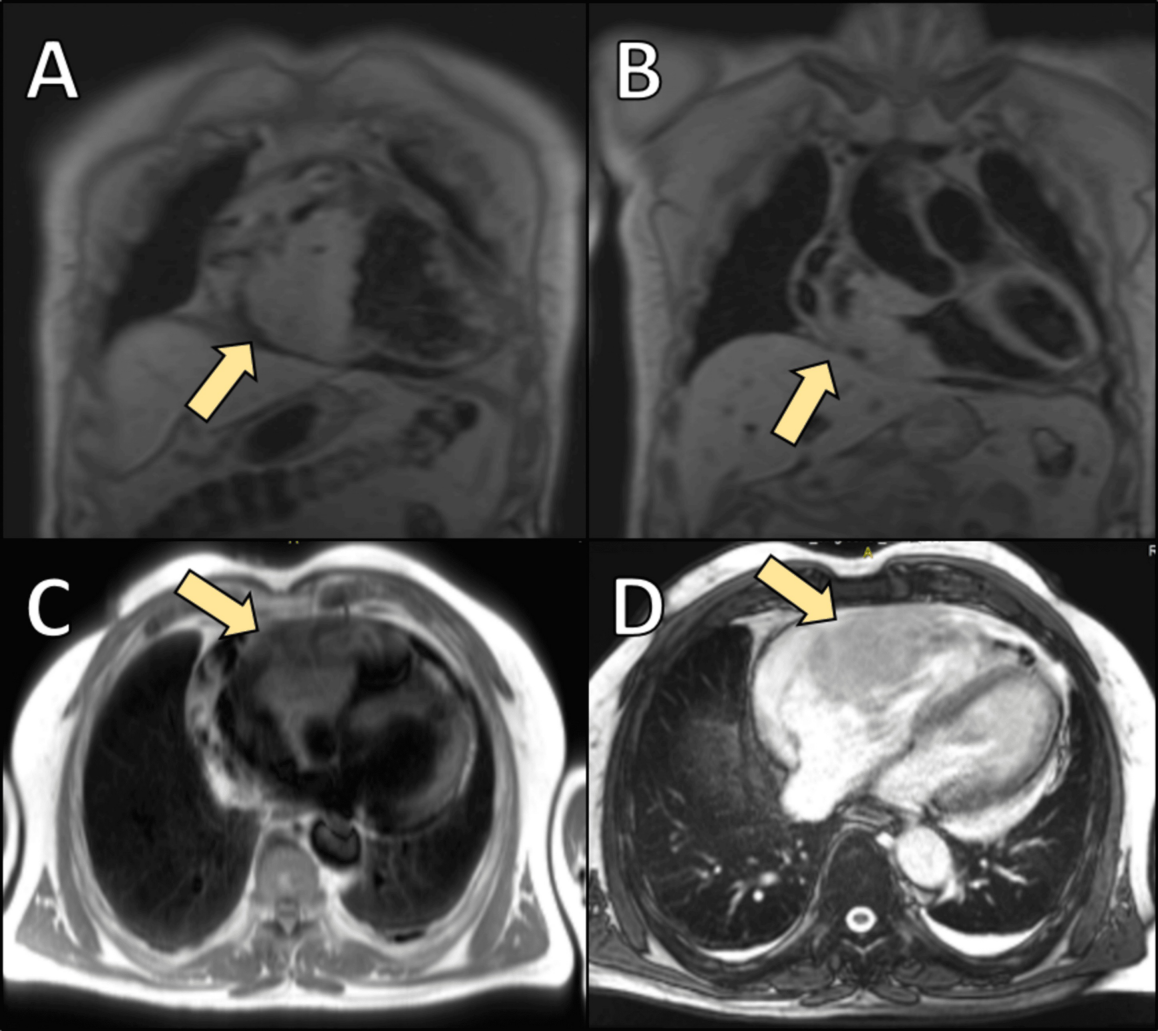
Magnetic resonance imaging (MRI) of a cardiac tumor in the right atrium and ventricle. (A) Coronal reconstruction of a T1-weighted sequence showing the distribution of the mass within the right atrium (arrows). (B) Coronal plane of a T1-weighted sequence highlighting the tumor's extension into the right ventricle. (C) Transverse plane of a T1-weighted sequence depicting the mass within the right ventricle. (D) Transverse cross-sectional plane of a T2-weighted sequence demonstrating the mass's extension and spatial orientation.

Initial 2D echocardiography revealed asymmetric and heterogeneous thickening of the right ventricular wall. A subsequent cardiac MRI showed that the left ventricle had normal dimensions and good systolic function, with an ejection fraction of 70%. However, a large mass was observed infiltrating the right ventricle wall, involving the tricuspid valve annulus, and extending into the right atrium (Figure 1). The mass measured approximately 10 cm in length and 8 cm in width, encroaching on a segment of the right coronary artery and exhibiting uneven contrast uptake, suggestive of malignancy originating from right ventricular structures.

**Figure 2:**
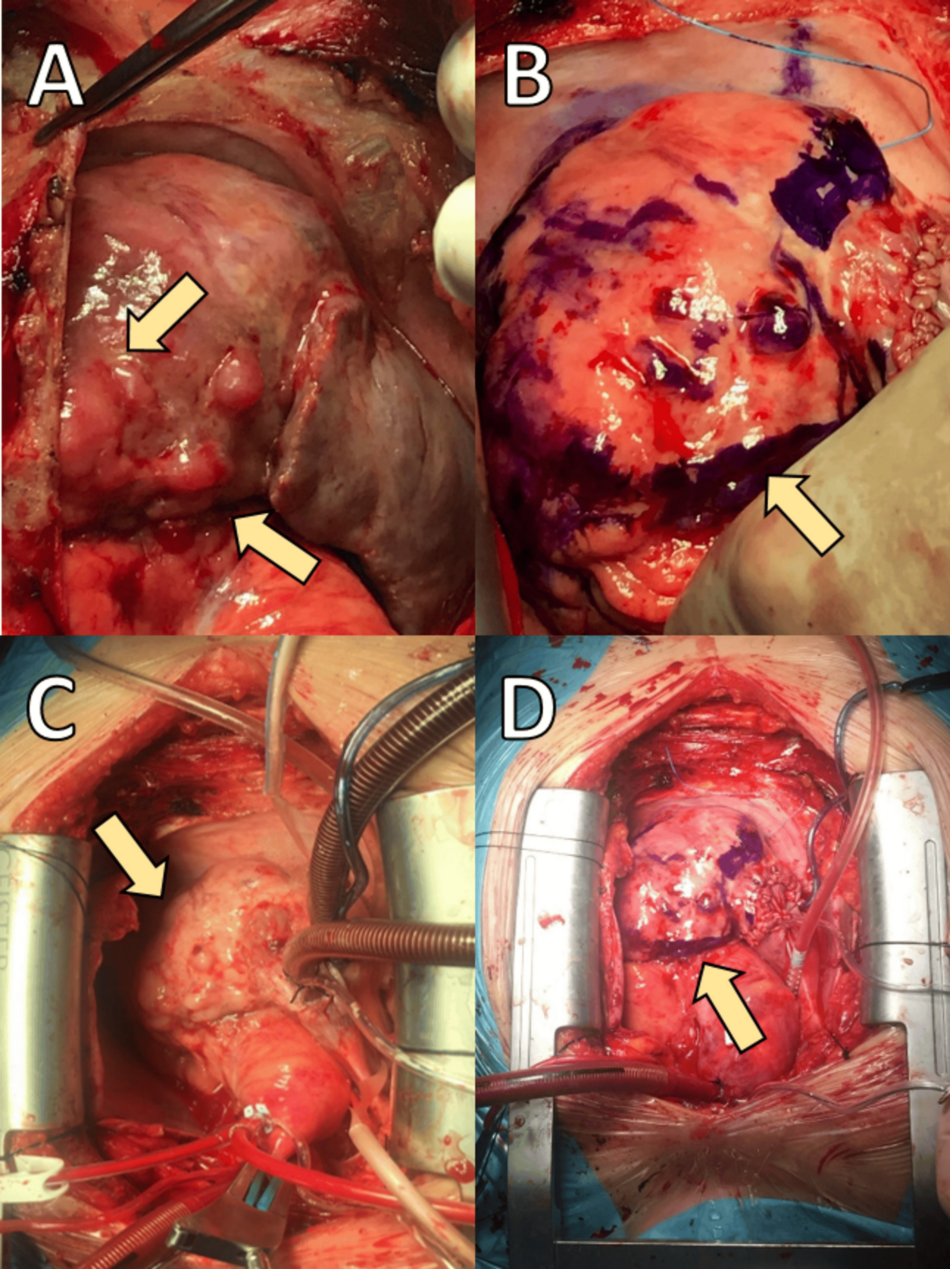
Intraoperative identification of the tumor in the right heart chambers (arrows). (A) Localization of the tumor in the right atrium and right ventricle (arrows), revealing a large, irregular mass extending from the right atrial wall and infiltrating adjacent right ventricular structures. (B) Tumor margins marked with blue dye (arrows) to delineate its extent. (C) Cardiopulmonary bypass is utilized to maintain systemic circulation during tumor resection. (D) The surgical field showing the exposed right heart chambers, highlighting the tumor's extensive involvement and its impact on surrounding cardiac structures.

**Figure 3:**
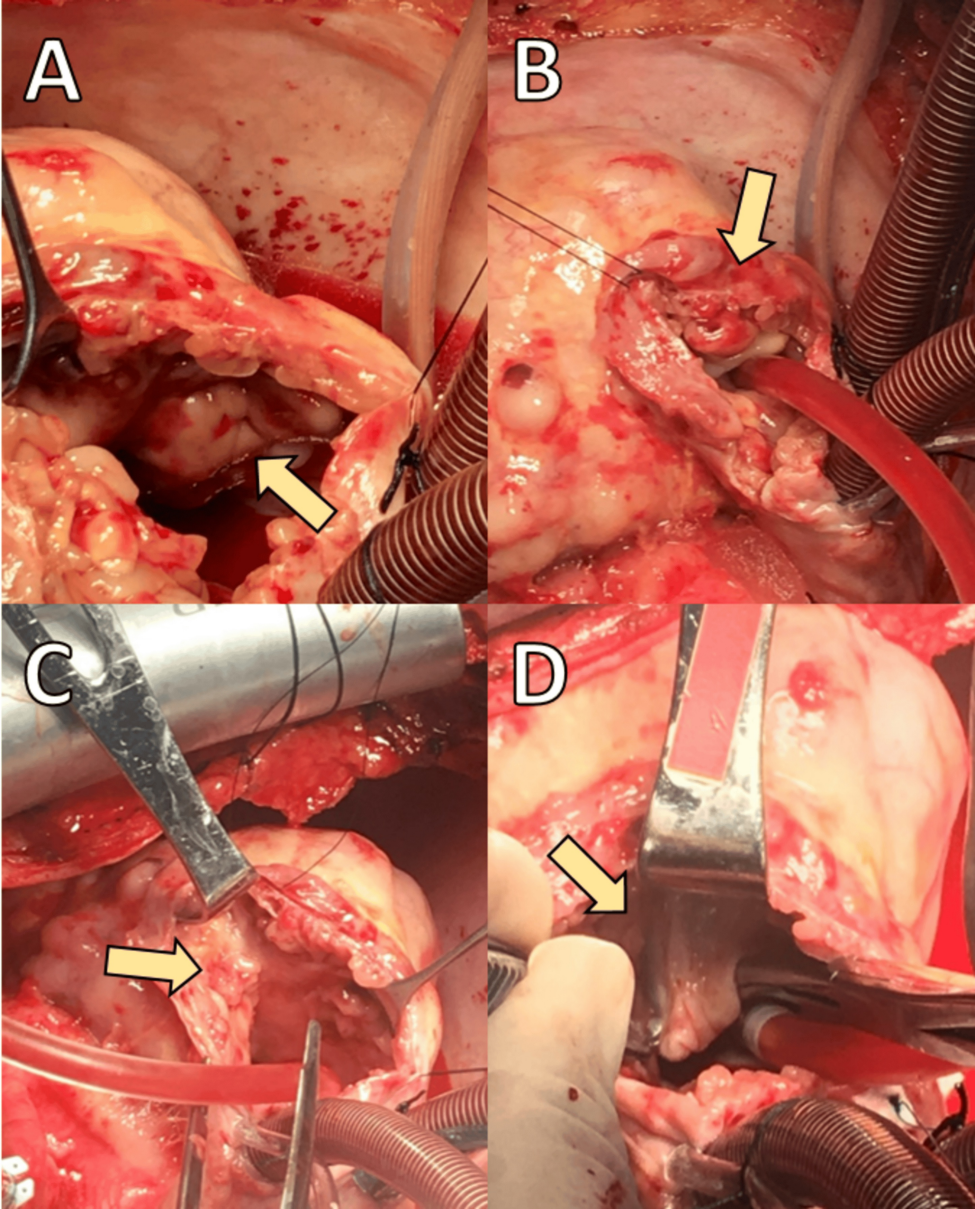
Surgical debulking and partial removal of the tumor from the right heart chambers. (A) Initial incision exposing the tumor mass infiltrating the roof of the right atrium (arrow). (B) Visualization of the extensive infiltration of the right atrial wall, characterized by irregular margins (arrow). (C) Deeper extension of the tumor within the right atrium (arrow), with careful dissection performed to separate the mass from surrounding cardiac structures while minimizing damage to the heart tissue. (D) Tumor expansion involving the tricuspid valve (arrow). Additional tumor tissue is meticulously separated from the tricuspid valve region under cardiopulmonary bypass to reduce the risk of valve obstruction. The final stages of debulking show visible portions of the tumor successfully removed, improving clearance around the tricuspid valve and right atrial structures.

Given the tumor's size, extent, and potential vascular involvement, the patient was scheduled for a critical palliative surgical intervention. The patient underwent tumor debulking surgery via a right atriotomy under total cardiopulmonary bypass (CPB). The CPB duration was approximately 93 minutes, with an aortic cross-clamp (ACC) time of 42 minutes. Intraoperatively, a large, irregularly shaped mass infiltrating the right atrium and ventricle, as well as the tricuspid valve annulus, was identified (Figure 2). The tumor extended into the subvalvular region and adjacent right coronary artery, with no evidence of complete coronary obstruction. The tumor margins were irregular, necessitating meticulous dissection to minimize damage to adjacent structures. Partial tumor resection was performed to alleviate obstruction at the tricuspid valve annulus and ensure improved blood flow dynamics. Tumor tissue was excised from the right atrial wall, the tricuspid valve annulus, and the subvalvular area of the right ventricle (Figure 3). Although a complete resection was not feasible due to extensive infiltration, debulking successfully reduced the mass burden.

Post-cardiopulmonary bypass, transesophageal echocardiography (TEE) revealed mild tricuspid regurgitation, which was attributed to residual valvular involvement. Left ventricular function was preserved, with a normal ejection fraction of 60% observed on TEE. There were no immediate complications related to the surgical procedure, and the patient was hemodynamically stable.

Histological examination revealed extensive infiltration by large, round-to-oval cells, raising a differential diagnosis of sarcoma versus lymphoma. Immunohistochemical analysis of the excised tissue subsequently confirmed the diagnosis of diffuse large B-cell non-Hodgkin lymphoma, classified as the germinal center B-cell (GCB) subtype.

Following stabilization, PET-CT staging demonstrated increased radiotracer uptake in an enlarged aortopulmonary lymph node (maximum standardized uptake value (SUVmax) = 17.5), the right ventricular mass (SUVmax = 20.6), and a right paracardiac lymph node (SUVmax = 8.9). Additionally, mild radiotracer uptake (SUVmax = 3.4) was observed in the posterior left second rib, likely post-traumatic.

Chemotherapy with CHOP (cyclophosphamide 750 mg/m², doxorubicin 50 mg/m², vincristine 1.4 mg/m², and prednisone 100 mg) was commenced on postoperative day 18. However, inadequate response and persistent thrombocytopenia led to the addition of rituximab, a CD20-targeting monoclonal antibody, followed by mycophenolate mofetil for refractory ITP. These therapies provided symptom and platelet count improvement, though intermittent thrombocytopenia episodes persisted. The patient was subsequently transferred to a local hospital to facilitate ongoing care.

Five months into treatment, the patient experienced febrile episodes, suspected to be due to sternal osteomyelitis, a postoperative complication. Empirical antibiotics, including vancomycin (20 mg/kg × 70 kg = 1400 mg IV every eight to 12 hours), resolved the symptoms. Follow-up MRI raised concerns about residual disease or chronic osteomyelitis. A PET-CT later showed mild radiotracer uptake around the tricuspid valve, suggestive of possible residual lymphoma activity, leading to the consideration of adjuvant radiotherapy targeting the tricuspid valve area.

Seven years post treatment, the patient remains in complete remission, asymptomatic, and enjoys a good quality of life. Cardiac and oncologic evaluations confirm disease stability, underscoring the successful long-term management of this complex PCL case.

## Discussion

PCL remains a rare but significant diagnostic challenge due to its nonspecific presentation and potential for extensive local invasion. In a retrospective study examining 94 cases of biopsy-confirmed cardiac involvement in non-Hodgkin lymphoma (NHL) reported in PubMed between 1990 and 2015, Gordon et al. observed that while cardiac manifestations in NHL are relatively uncommon clinically, autopsy data suggest that up to 20% of NHL cases reveal cardiac involvement. Furthermore, cardiac NHL accounts for approximately 2% of all cardiac malignancies [4]. Cardiac involvement in NHL is most frequently of B-cell origin, with DLBCL being the predominant subtype. The clinical presentation of PCL varies based on the anatomical site of involvement, though it commonly affects the right side of the heart. Typical symptoms align with those of heart failure and may include pericardial effusions and atrioventricular nodal block, depending on the extent and location of the disease [5]. It is noteworthy that approximately 20% of patients present with acute heart failure as the initial manifestation of the disease [6].

These tumors, due to their atypical symptomatology, are primarily incidental findings during imaging studies. Initial information regarding the tumor's location, size, and impact on the patient's hemodynamic status is typically obtained through 2D echocardiography. However, the preferred imaging modality is cardiac MRI, as it offers the highest sensitivity and provides a more accurate evaluation of the tumor's characteristics [7]. PET-CT is effective in evaluating systemic invasion and the metabolic activity of the tumor, while cytological examination of the tumor can confirm the presence of lymphoma cells, aiding in the diagnosis [8].

Chemotherapy and surgery are the primary therapeutic options for these patients. Surgical intervention not only alleviates symptoms caused by obstructive phenomena but also facilitates definitive tumor identification through pathological analysis. The chemotherapy regimen most commonly employed in these cases is CHOP [9].

The prognosis for patients with PCL remains poor, with over 60% of patients succumbing to the disease within two months of diagnosis. Reported survival durations range from 1.5 to 26.5 months, with an estimated median survival of approximately 12 months [6].

This case exemplifies the unique presentation of PCL as a right ventricular mass with associated immune thrombocytopenia managed through a combination of surgery, chemotherapy, immunotherapy, and supportive care. DLBCL is the most common histological subtype associated with PCL and often responds to the CHOP regimen [5]. However, this patient's initial response to CHOP was suboptimal, necessitating the addition of rituximab, a monoclonal antibody targeting CD20, which has become standard in DLBCL treatment.

Thrombocytopenia is an extremely rare finding in association with PCL as well as other primary cardiac tumors and, when present, can significantly increase diagnostic complexity and patient morbidity. To our knowledge, only a few cases have been described in the literature to date. The pathogenesis of thrombocytopenia in cardiac tumors may involve mechanical platelet shearing due to turbulent, obstructed cardiac blood flow, along with platelet consumption through microthrombi formation within the tumor itself [10]. Thrombocytopenia is attributed to either increased peripheral destruction of platelets or decreased production in the bone marrow [11]. As in the case identified after an extensive review of the literature, our patient had no prior history or risk factors for chronic liver disease, no splenomegaly was identified, and the thrombocytopenia was attributed to one of the aforementioned mechanisms. Furthermore, as evidenced by the patient's laboratory results, platelet counts increased immediately following the surgical removal of the tumor, supporting our hypothesis that the thrombocytopenia was due to one of the mechanisms described above. The patient’s refractory thrombocytopenia also necessitated treatment with mycophenolate mofetil, which ultimately provided symptomatic relief and improved platelet counts. Febrile episodes five months post diagnosis were initially concerning for lymphoma recurrence; however, they were attributed to sternal osteomyelitis, a postoperative complication, which was successfully treated with antibiotics. This case demonstrates the need for a multidisciplinary approach to managing PCL, particularly when complicated by immune-mediated hematologic disorders and infectious concerns.

This patient's case was further complicated by persistent inflammatory findings on PET-CT, which raised concerns for residual disease in the right ventricle and tricuspid valve area. While additional radiotherapy was considered, the decision must weigh the potential benefits against the risks of cardiac toxicity. Overall, this case underlines the importance of a patient-centered, multidisciplinary approach in managing PCL, particularly when complicated by immune-mediated conditions and potential infection risks. The successful long-term outcome achieved in this patient highlights the need for vigilant follow-up and individualized treatment adaptations, which are essential for optimizing prognosis in such rare and complex malignancies. Continued study and reporting of PCL cases will further enhance our understanding and refine therapeutic strategies, ultimately contributing to improved outcomes for future patients with this challenging diagnosis.

## Conclusions

This case illustrates the complex presentation and management of PCL, emphasizing the importance of a tailored, multidisciplinary approach. Despite an initial suboptimal response to chemotherapy and subsequent complications, the patient achieved remission and has remained disease-free over a seven-year follow-up. Continued surveillance is warranted in PCL cases due to the potential for late recurrence or complications related to initial treatments. This report contributes to the limited literature on PCL and highlights the evolving management strategies, including advancements in diagnostic modalities and tailored chemotherapy regimens, for achieving long-term remission in this rare malignancy.
